# Supplementary material for: Characterization, Comparative Analysis and Phylogenetic Implications of Mitogenomes of Fulgoridae (Hemiptera: Fulgoromorpha)
Source: Genes (Basel). 2021 Jul 30;12(8):1185. doi: 10.3390/genes12081185 (PMC8394797; doi:10.3390/genes12081185)
Supplement: Supplementary file 1 [file genes-12-01185-s001.zip › genes-1277800-supplementary/Supplementary Materials/Supplementary Tables S1-S5.pdf]

Table S1. Mitogenomic organization of nine Fulgoridae mitochondrial genomes.
Dichoptera sp. (*D.*); *Limois* sp. (*L.*); *Neoalcathous huangshanana* (*N.*).

Gene	Position		Size (bp)	Codon		Strand
	From	To		Start	Stop	
D./L./N.						
trnI	1/1/1	63/65/63	63/65/63			+/+/+
trnQ	66/72/72	134/140/414	69/69/70			-/-/-
trnM	134/140/141	197/204/204	64/65			+/+/+
nad2	198/205/205	1163/1167/1143	966/963/939	ATT/ATT/ATT	TAA/TAA/TAA	+/+/+
trnW	1162/1174/1202	1229/1236/1269	68/63/68			+/+/+
trnC	1222/1229/1262	1287/1290/1329	66/62/68			-/-/-
trnY	1290/1293/1332	1351/1356/1394	62/64/63			-/-/-
cox1	1357/1360/1409	2895/2899/2947	1539/1539/1539	ATG/ATG/ATG	TAA/TAA/TAA	+/+/+
trnL2	2891/2895/2943	2958/2959/3009	68/65/67			+/+/+
cox2	2959/2960/3010	3630/3631/3684	672/672/675	ATA/ATA/ATA	TAA/TAA/TAA	+/+/+
trnK	3637/3639/3694	3708/3709/3764	72/71/71			+/+/+
trnD	3713/3710/3765	3774/3772/3827	62/63/63			+/+/+
atp8	3775/3773/3828	3930/3931/3986	156/159/159	ATT/ATT/ATC	TAA/TAA/TAA	+/+/+
atp6	3924/3925/3980	4575/4576/4631	652/652/652	ATG/ATG/ATG	T/T/T	+/+/+
cox3	4576/4577/4632	5356/5359/5414	781/783/783	ATG/ATG/ATG	T/TAA/TAA	+/+/+
trnG	5357/5361/5422	5417/5425/5485	61/65/64			+/+/+
nad3	5418/5426/5486	5765/5773/5833	348/348/348	ATT/ATT/ATA	TAA/TAA/TAA	+/+/+
trnA	5766/5795/5837	5830/5858/5900	65/64/64			+/+/+
trnR	5831/5861/5901	5891/5922/5960	61/62/60			+/+/+
trnN	5894/5828/5963	5962/5991/6029	69/64/67			+/+/+
trnS1	5962/5991/6030	6021/6048/6087	60/58/67			+/+/+
trnE	6021/6050/6092	6076/6113/6152	56/64/61			+/+/+
trnF	6083/6112/6151	6149/6176/6215	67/65/65			-/-/-
nad5	6144/6167/6206	7805/7837/7879	1662/1671/1674	ATT/ATG/ATA	TAA/TAG/TAG	-/-/-
trnH	7817/7838/7893	7879/7899/7955	63/62/63			-/-/-
nad4	7870/7890/7937	9204/9224/9268	1335/1335/1332	ATG/ATG/ATT	TAG/TAG/TAA	-/-/-
nad4L	9198/9218/9274	9473/9493/9549	276/276/276	ATG/ATG/ATG	TAA/TAG/TAA	-/-/-
trnT	9480/9502/9569	9544/9567/9632	65/66/64			+/+/+
trnP	9550/9575/9636	9613/9638/9698	64/64/61			-/-/-
nad6	9618/9646/9698	10112/10140/10192	495/495/495	ATA/ATT/ATT	TAA/TAA/TAA	+/+/+
cytb	10119/10133/10203	11240/11251/11327	1122/1119/1125	ATG/ATG/ATG	TAA/TAA/TAA	+/+/+
trnS2	11240/11251/11353	11302/11312/11414	63/62/62			+/+/+
nad1	11305/11306/11426	12247/12250/12358	943/945/933	ATG/ATG/ATG	T/TAA/TAA	-/-/-
trnL1	12249/12252/12360	12314/12314/12422	66/64/63			-/-/-
rrnL	12315/12315/12423	13532/13527/13635	1218/1213/1213			-/-/-
trnV	13533/13528/13636	13600/13591/13696	68/64/61			-/-/-
rrnS	13601/13592/13697	14334/14323/14428	734/732/732			-/-/-
A +T-rich region	14335/14324/14429	15803/15957/16510	1469/1634/2082			+/+/+

Table S2. Mitogenomic organization of nine Fulgoridae mitochondrial genomes.
Penthicodes atomaria (*Pea.*); *Penthicodes caja* (*Pec.*); *Penthicodes variegata* (*Pev.*).

Gene	Position		Size (bp)	Codon		Strand
	From	To		Start	Stop	
Pea./Pec./Pev.						
trnI	1/1/1	64/64/64	64/64/64			+/+/+
trnQ	81/77/77	149/145/145	69/69/69			-/-/-
trnM	149/145/145	212/210/210	64/66/66			+/+/+
nad2	219/217/211	1184/1182/1179	966/966/969	ATA/ATA/ATT	TAA/TAA/TAA	+/+/+
trnW	1194/1188/1228	1256/1251/1291	63/64/64			+/+/+
trnC	1249/1244/1284	1311/1303/1344	63/60/61			-/-/-
trnY	1318/1311/1356	1379/1373/1420	62/63/65			-/-/-
cox1	1385/1390/1426	2923/2925/2961	1539/1536/1536	ATG/ATG/ATG	TAA/TAA/TAA	+/+/+
trnL2	2924/2929/2964	2987/2995/3027	64/67/64			+/+/+
cox2	2988/2996/3028	3659/3670/3702	672/675/675	ATA/ATA/ATA	TAA/TAA/TAA	+/+/+
trnK	3665/3673/3705	3734/3743/3774	70/71/70			+/+/+
trnD	3735/3744/3775	3796/3808/3840	62/65/66			+/+/+
atp8	3797/3809/3841	3958/3964/3996	162/156/156	ATT/ATT/ATT	TAA/TAA/TAA	+/+/+
atp6	3952/3958/3990	4603/4609/4641	652/652/652	ATG/ATG/ATG	T/T/T	+/+/+
cox3	4604/4610/4642	5386/5392/5424	783/783/783	ATG/ATG/ATG	TAA/TAA/TAA	+/+/+
trnG	5407/5414/5441	5469/5477/5504	63/64/64			+/+/+
nad3	5470/5478/5505	5817/5825/5852	348/348/348	ATT/ATT/ATT	TAG/TAA/TAA	+/+/+
trnA	5816/5843/5876	5883/5907/5943	68/65/68			+/+/+
trnR	5887/5913/5968	5949/5978/6030	63/66/63			+/+/+
trnN	5954/5987/6041	6016/6053/6105	63/67/65			+/+/+
trnS1	6017/6054/6106	6073/6113/6163	57/60/58			+/+/+
trnE	6076/6116/6166	6137/6180/6232	62/65/67			+/+/+
trnF	6136/6179/6237	6199/6243/6302	64/65/66			-/-/-
nad5	6194/6241/6300	7867/7912/7971	1674/1672/1672	ATG/ATG/ATG	TAG/T/T	-/-/-
trnH	7868/7898/7972	7930/7961/8034	63/64/63			-/-/-
nad4	7921/7967/8082	9261/9301/9359	1341/1335/1335	ATG/ATG/ATT	TAG/TAG/TAG	-/-/-
nad4L	9255/9307/9359	9530/9582/9634	276/276/276	ATG/ATG/ATG	TAG/TAA/TAA	-/-/-
trnT	9545/9590/9643	9611/9655/9776	67/66/64			+/+/+
trnP	9614/9663/9713	9676/9726/9776	63/64/64			-/-/-
nad6	9678/9758/9778	10181/10258/10281	504/501/504	ATA/ATC/ATT	TAA/TAA/TAA	+/+/+
cytb	10174/10239/10274	11292/11357/11395	1119/1119/1122	ATG/ATG/ATG	TAG/TAA/TAG	+/+/+
trnS2	11291/11343/11394	11352/11409/11456	62/67/63			+/+/+
nad1	11346/11418/11450	12269/12353/12370	924/936/921	ATA/ATG/ATT	TAA/TAA/TAA	-/-/-
trnL1	12298/12340/12402	12360/12403/12465	63/64/64			-/-/-
rrnL	12361/12404/12466	13575/13624/13689	1215/1221/1224			-/-/-
trnV	13576/13625/13690	13635/13686/13751	60/62/62			-/-/-
rrnS	13636/13687/13752	14365/14442/14487	730/756/736			-/-/-
A +T-rich region	14366/14443/14488	16093/16040/15814	1728/1598/1327			+/+/+

Table S3. Mitogenomic organization of nine Fulgoridae mitochondrial genomes.
Pyrops clavatus (*Pyc.*); *Pyrops lathburii* (*Pyl.*) and *Pyrops spinolae* (*Pys.*).

Gene	Position		Size (bp)	Codon		Strand
	From	To		Start	Stop	
Pyc./Pyl./Pys.						
trnI	1/1/1	64/64/64	64/64/64			+/+/+
trnQ	71/71/71	140/140/140	70/70/70			-/-/-
trnM	140/141/140	203/204/203	64/64/64			+/+/+
nad2	210/205/204	1178/1170/1172	969/966/969	ATA/ATT/ATT	TAA/TAA/TAA	+/+/+
trnW	1211/1193/1201	1280/1262/1269	70/70/69			+/+/+
trnC	1273/1255/1262	1337/1322/1329	65/68/68			-/-/-
trnY	1338/1323/1330	1400/1385/1392	63/63/63			-/-/-
cox1	1408/1397/1407	2946/2935/2945	1539/1539/1539	ATG/ATG/ATG	TAA/TAA/TAA	+/+/+
trnL2	2942/2931/2941	3008/2996/3006	67/66/66			+/+/+
cox2	3009/2997/3007	3683/3671/3681	675/675/675	ATA/ATA/ATA	TAA/TAA/TAA	+/+/+
trnK	3686/3674/3684	3756/3744/3754	71/71/71			+/+/+
trnD	3758/3745/3755	3820/3807/3817	63/63/63			+/+/+
atp8	3821/3808/3818	3976/3966/3979	156/159/162	ATT/ATT/ATC	TAA/TAA/TAA	+/+/+
atp6	3970/3960/3973	4621/4611/4624	652/652/652	ATG/GTG/ATG	T/T/T	+/+/+
cox3	4622/4612/4625	5404/5394/5407	783/783/783	ATG/ATG/ATG	TAA/TAA/TAA	+/+/+
trnG	5413/5401/5408	5474/5463/5471	62/63/64			+/+/+
nad3	5475/5464/5472	5822/5811/5819	348/348/348	ATA/ATT/ATT	TAA/TAA/TAG	+/+/+
trnA	5850/5839/5846	5915/5909/5912	66/71/67			+/+/+
trnR	5921/5916/5920	5982/5979/5981	62/61/62			+/+/+
trnN	5985/5979/5984	6048/6043/6049	64/65/66			+/+/+
trnS1	6048/6043/6049	6106/6101/6108	59/59/60			+/+/+
trnE	6112/6105/6112	6172/6165/6173	61/61/62			+/+/+
trnF	6171/6164/6172	6234/6227/6235	64/64/64			-/-/-
nad5	6229/6218/6221	7902/7888/7894	1674/1671/1674	ATA/ATC/ATA	TAG/TAG/TAA	-/-/-
trnH	7916/7905/7908	7975/7965/7968	60/61/61			-/-/-
nad4	7966/7956/7959	9300/9290/9293	1335/1335/1335	ATG/ATG/ATG	TAG/TAG/TAG	-/-/-
nad4L	9294/9284/9287	9566/9559/9562	273/273/276	ATT/ATG/ATG	TAG/TAA/TAG	-/-/-
trnT	9608/9600/9599	9671/9669/9666	64/70/68			+/+/+
trnP	9677/9673/9670	9737/9733/9731	61/61/62			-/-/-
nad6	9739/9735/9733	10233/10229/10227	495/495/495	ATT/ATT/ATC	TAA/TAA/TAA	+/+/+
cytb	10249/10245/10243	11367/11363/11361	1119/1119/1119	ATA/ATA/ATA	TAA/TAA/TAA	+/+/+
trnS2	11367/11363/11361	11428/11424/11422	62/62/62			+/+/+
nad1	11439/11423/11432	12371/12364/12364	933/942/933	ATG/GTG/ATG	TAA/TAA/TAA	-/-/-
trnL1	12373/12366/12366	12435/12428/12428	63/63/63			-/-/-
rrnL	12436/12429/12429	13645/13640/13640	1210/1212/1212			-/-/-
trnV	13646/13641/13641	13705/13701/13701	60/61/61			-/-/-
rrnS	13706/13702/13702	14443/14432/14433	738/731/732			-/-/-
A +T-rich region	14444/14433/14434	16054/16104/16028	1611/1672/1595			+/+/+

Table S4. Start and stop codons usage of nine Fulgoridae mitogenomes.

Gene	Start Codon / Stop Codon								
	D.	*L.*	*N.*	*Pea.*	*Pec.*	*Pev.*	*Pyc.*	*Pyl.*	*Pys.*
nad2	ATT/TAA	ATT/TAA	ATT/TAA	ATA/TAA	ATA/TAA	ATT/TAA	ATA/TAA	ATT/TAA	ATT/TAA
cox1	ATG/TAA	ATG/TAA	ATG/TAA	ATG/TAA	ATG/TAA	ATG/TAA	ATG/TAA	ATG/TAA	ATG/TAA
cox2	ATA/TAA	ATA/TAA	ATA/TAA	ATA/TAA	ATA/TAA	ATA/TAA	ATA/TAA	ATA/TAA	ATA/TAA
atp8	ATT/TAA	ATT/TAA	ATC/TAA	ATT/TAA	ATT/TAA	ATT/TAA	ATT/TAA	ATT/TAA	ATC/TAA
atp6	ATG/T	ATG/T	ATG/T	ATG/T	ATG/T	ATG/T	ATG/T	GTG/T	ATG/T
cox3	ATG/TAA	ATG/TAA	ATG/T	ATG/TAA	ATG/TAA	ATG/TAA	ATG/TAA	ATG/TAA	ATG/TAA
nad3	ATT/TAA	ATT/TAA	ATA/TAA	ATT/TAG	ATT/TAA	ATT/TAA	ATA/TAA	ATT/TAA	ATT/TAG
nad5	ATT/TAA	ATG/TAG	ATA/TAG	ATG/TAG	ATG/T	ATG/T	ATA/TAG	ATC/TAG	ATA/TAA
nad4	ATG/TAG	ATG/TAG	ATT/TAA	ATG/TAG	ATG/TAG	ATT/TAG	ATG/TAG	ATG/TAG	ATG/TAG
nad4L	ATG/TAA	ATG/TAA	ATG/TAG	ATG/TAG	ATG/TAA	ATG/TAA	ATT/TAG	ATG/TAA	ATG/TAG
nad6	ATA/TAA	ATT/TAA	ATT/TAA	ATA/TAA	ATC/TAA	ATT/TAA	ATT/TAA	ATT/TAA	ATC/TAA
cytb	ATG/TAA	ATG/TAA	ATG/TAG	ATG/TAA	ATG/TAA	ATG/TAG	ATA/TAA	ATA/TAA	ATA/TAA
nad1	ATG/TAA	ATG/TAA	ATG/T	ATA/TAA	ATG/TAA	ATT/TAA	ATG/TAA	GTG/TAA	ATG/TAA

Table S5. Best partitioning scheme and models used in this study.

Data matrix	Subset Partitions	Best Model
ML	P1: (*cox3*_codon1, *atp6*_codon1, *cox2*_codon1, *cytb*_codon1)	GTR+I+G
	P2: (*cox3*_codon2, *atp6*_codon2, *cox2*_codon2, *cytb*_codon2)	TVM+I+G
	P3: (*nad6*_codon3, *atp6*_codon3)	TRN+G
	P4: (*nad2*_codon1, *nad3*_codon1, *nad6*_codon1, *atp8*_codon1)	TIM+I+G
	P5: (*nad2*_codon2, *nad3*_codon2, *atp8*_codon2, *nad6*_codon2)	TVM+I+G
	P6: (*atp8*_codon3, *cox2*_codon3, *cox3*_codon3, *nad3*_codon3)	TIM+G
	P7: (*cox1*_codon1)	GTR+I+G
	P8: (*cox1*_codon2)	TVM+I+G
	P9: (*cox1*_codon3, *cytb*_codon3)	GTR+I+G
	P10: (*nad1*_codon1, *nad4L*_codon1, *nad5*_codon1, *nad4*_codon1)	GTR+I+G
	P11: (*nad4L*_codon2, *nad1*_codon2, *nad5*_codon2, *nad4*_codon2)	GTR+I+G
	P12: (*nad1*_codon3)	HKY+G
	P13: (*nad2*_codon3)	TIM+G
	P14: (*nad5*_codon3, *nad4L*_codon3, *nad4*_codon3)	HKY+G
	P15: (*rrnL*, *rrnS*)	GTR+I+G
BI	P1: (*cox3*_codon1, *atp6*_codon1, *cox2*_codon1, *cytb*_codon1)	GTR+I+G
	P2: (*cox2*_codon2, *cytb*_codon2, *cox3*_codon2, *atp6*_codon2)	GTR+I+G
	P3: (*nad6*_codon3, *atp6*_codon3, *atp8*_codon3)	GTR+G
	P4: (*nad2*_codon1, *atp8*_codon1, *nad3*_codon1, *nad6*_codon1)	GTR+I+G
	P5: (*nad2*_codon2, *nad3*_codon2, *atp8*_codon2, *nad6*_codon2)	GTR+I+G
	P6: (*cox1*_codon1)	GTR+I+G
	P7: (*cox1*_codon2)	GTR+I+G
	P8: (*cytb*_codon3, *cox1*_codon3)	GTR+I+G
	P9: (*nad3*_codon3, *cox2*_codon3, *cox3*_codon3)	GTR+G
	P10: (*nad1*_codon1, *nad5*_codon1, *nad4*_codon1, *nad4L*_codon1)	GTR+I+G
	P11: (*nad4L*_codon2, *nad1*_codon2, *nad5*_codon2, *nad4*_codon2)	GTR+I+G
	P12: (*nad1*_codon3)	HKY+G
	P13: (*nad2*_codon3)	GTR+G
	P14: (*nad5*_codon3, *nad4*_codon3, *nad4L*_codon3)	HKY+G
	P15: (*rrnL*, *rrnS*)	GTR+I+G
