# Supplementary figures and images for: Characterization, Comparative Analysis and Phylogenetic Implications of Mitogenomes of Fulgoridae (Hemiptera: Fulgoromorpha)
Source: Genes (Basel). 2021 Jul 30;12(8):1185. doi: 10.3390/genes12081185 (PMC8394797; doi:10.3390/genes12081185)

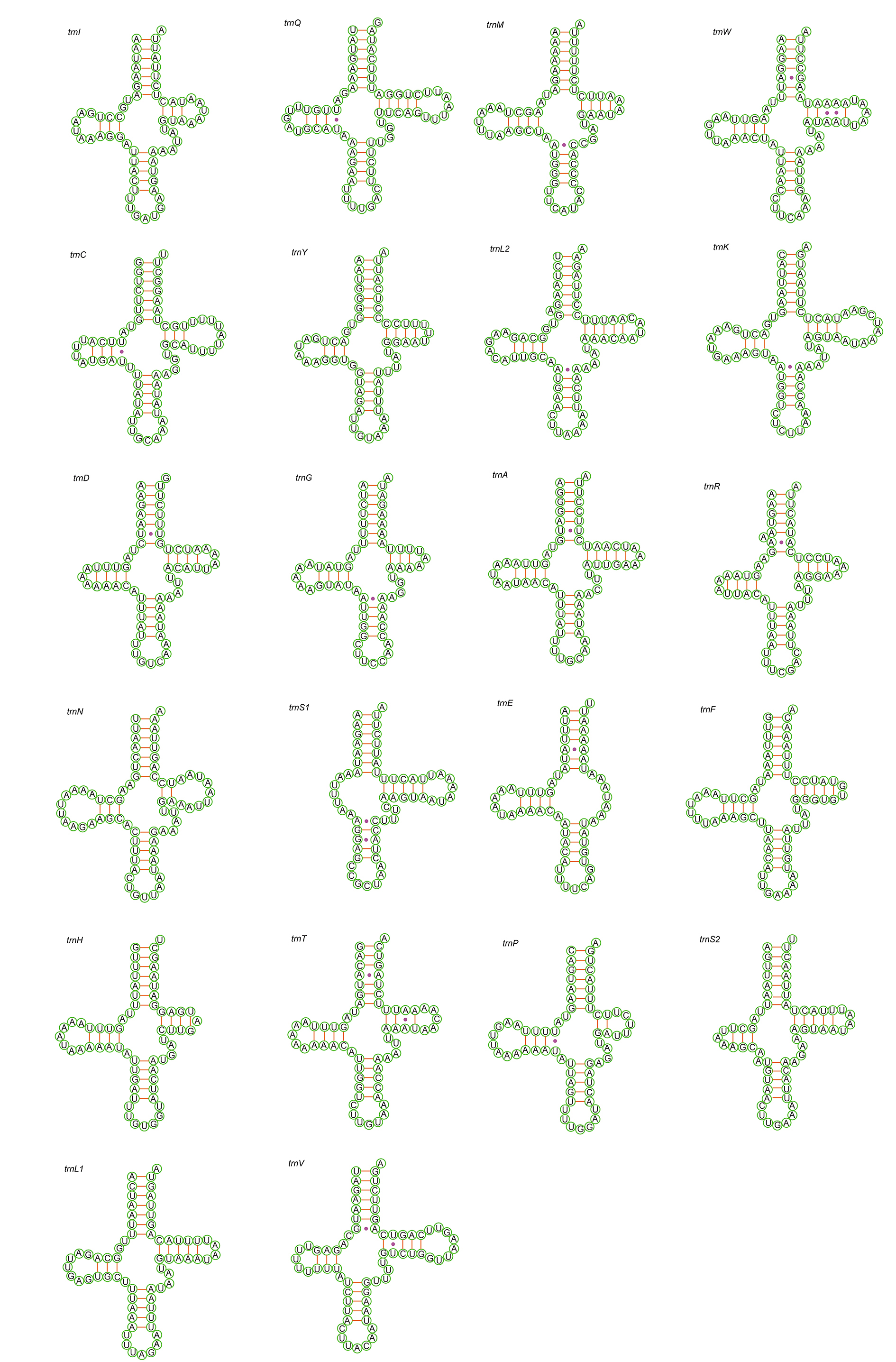

Supplement: Supplementary file 1 [file genes-12-01185-s001.zip › genes-1277800-supplementary/Supplementary Materials/Fig. S1 tRNA Dichoptera sp.jpg]

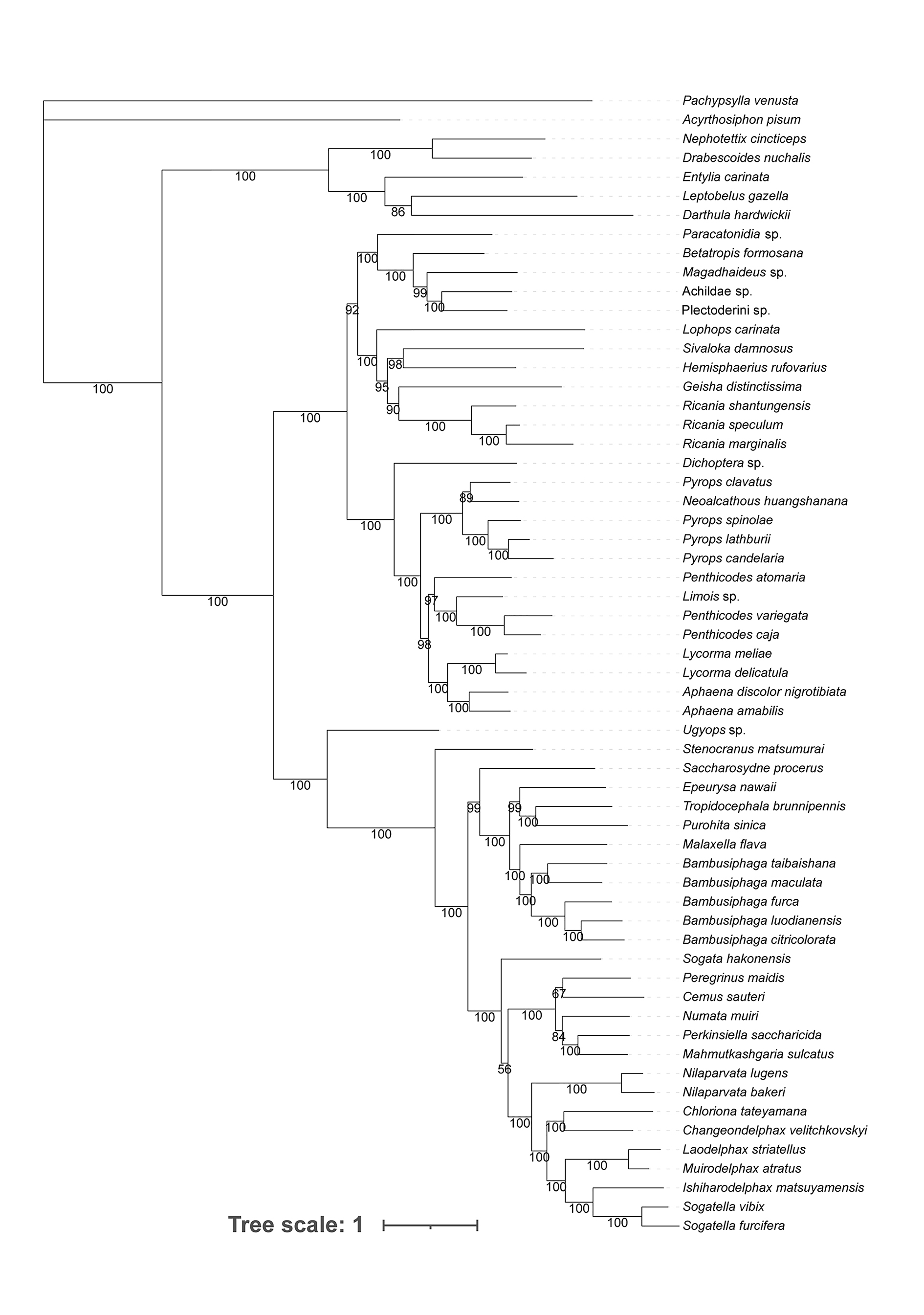

Supplement: Supplementary file 1 [file genes-12-01185-s001.zip › genes-1277800-supplementary/Supplementary Materials/Fig. S10 Phylogenetic tree ML_PCG123.jpg]

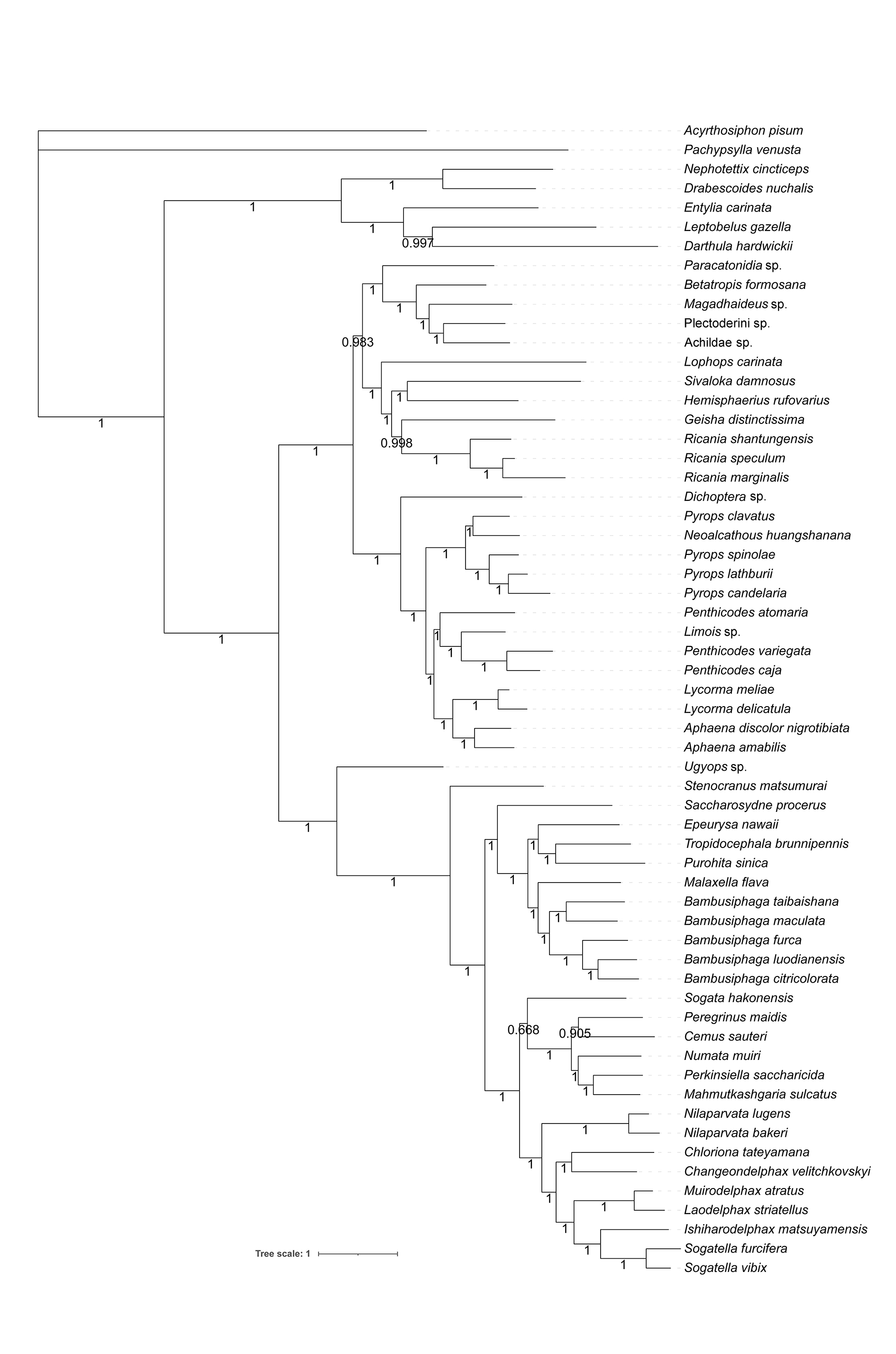

Supplement: Supplementary file 1 [file genes-12-01185-s001.zip › genes-1277800-supplementary/Supplementary Materials/Fig. S11 Phylogenetic tree BI_PCG123.jpg]

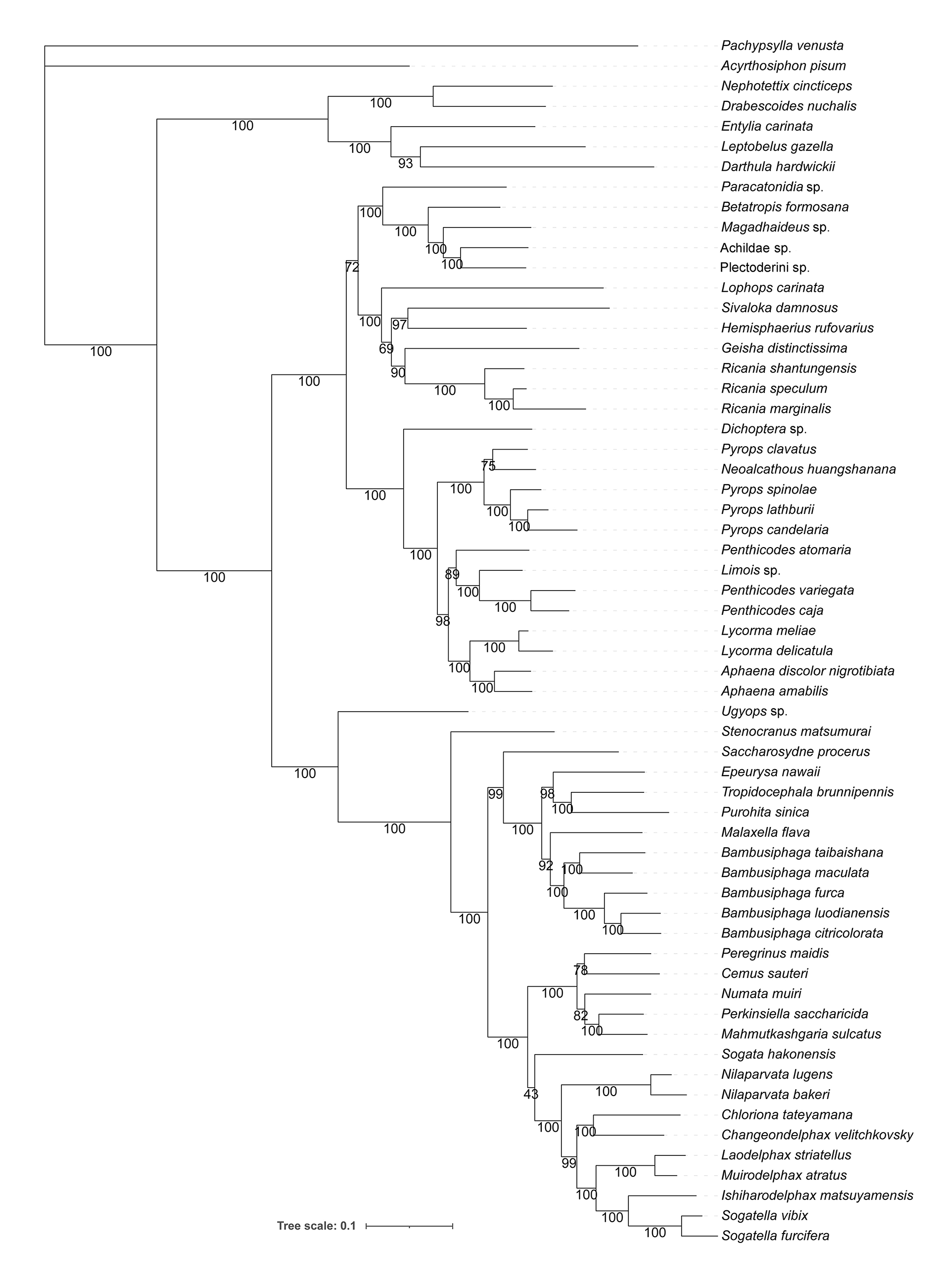

Supplement: Supplementary file 1 [file genes-12-01185-s001.zip › genes-1277800-supplementary/Supplementary Materials/Fig. S12 Phylogenetic tree ML_PCG12R.jpg]

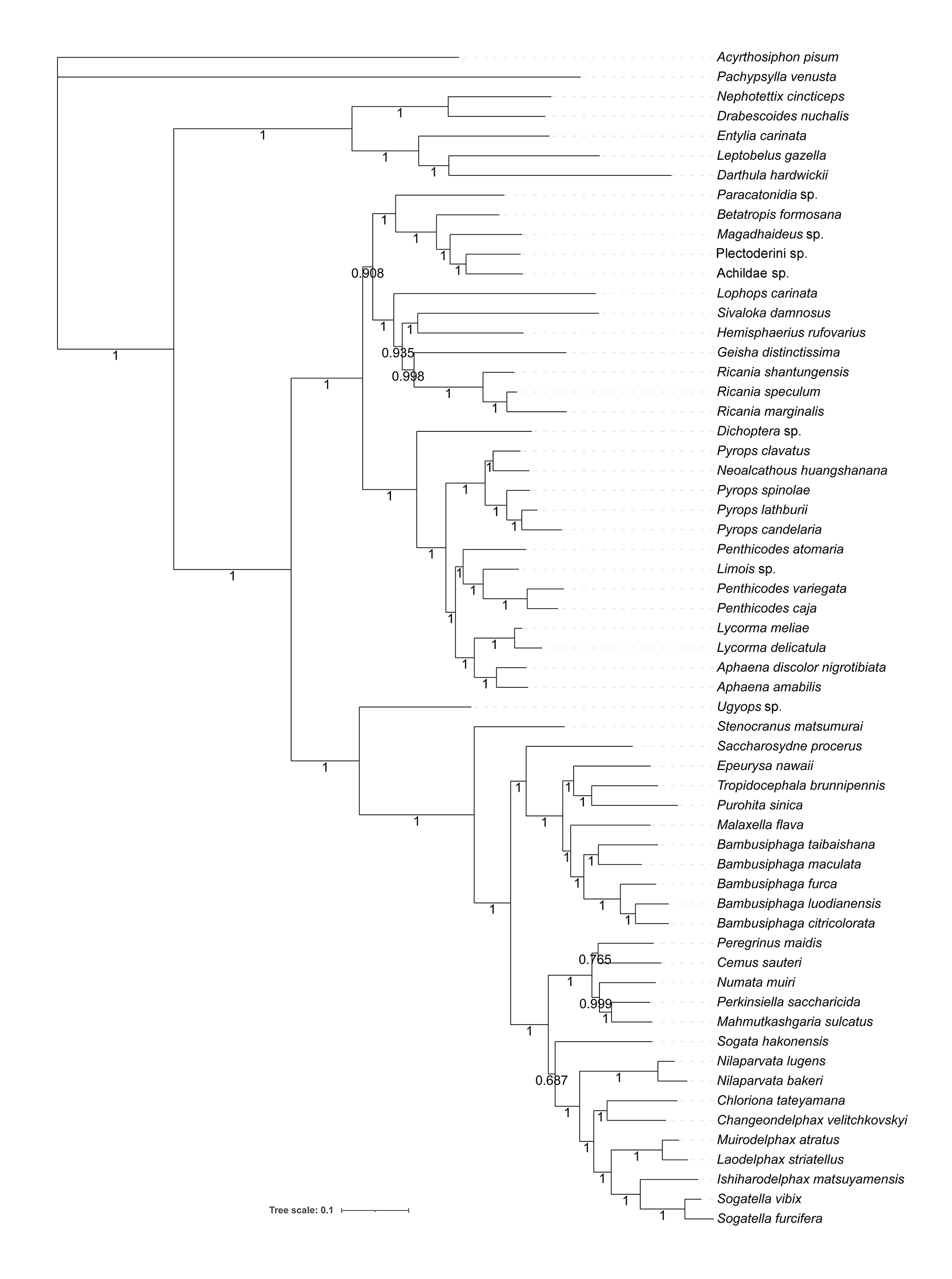

Supplement: Supplementary file 1 [file genes-12-01185-s001.zip › genes-1277800-supplementary/Supplementary Materials/Fig. S13 Phylogenetic tree BI_PCG12R.jpg]

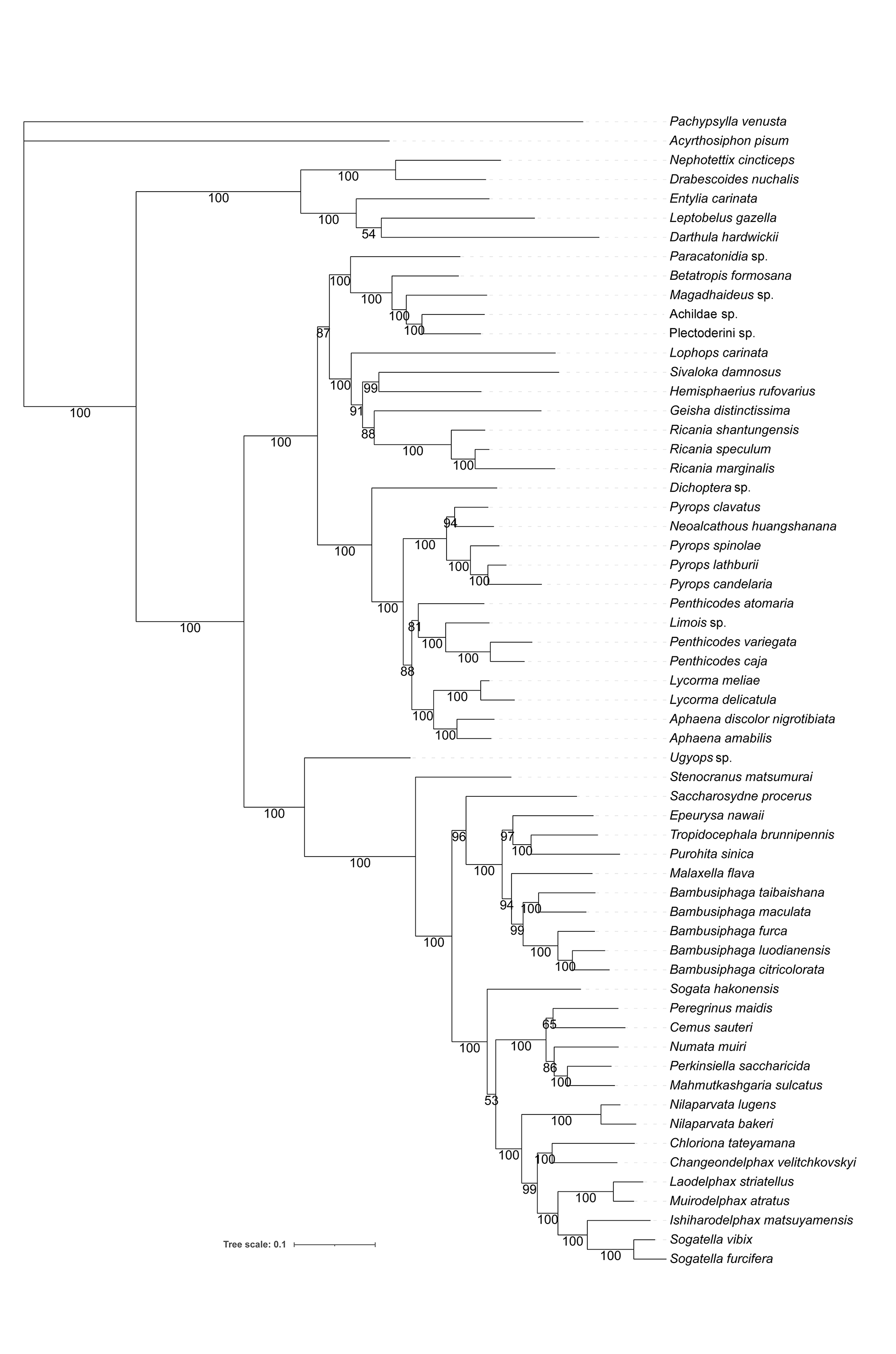

Supplement: Supplementary file 1 [file genes-12-01185-s001.zip › genes-1277800-supplementary/Supplementary Materials/Fig. S14 Phylogenetic tree ML_PCG12.jpg]

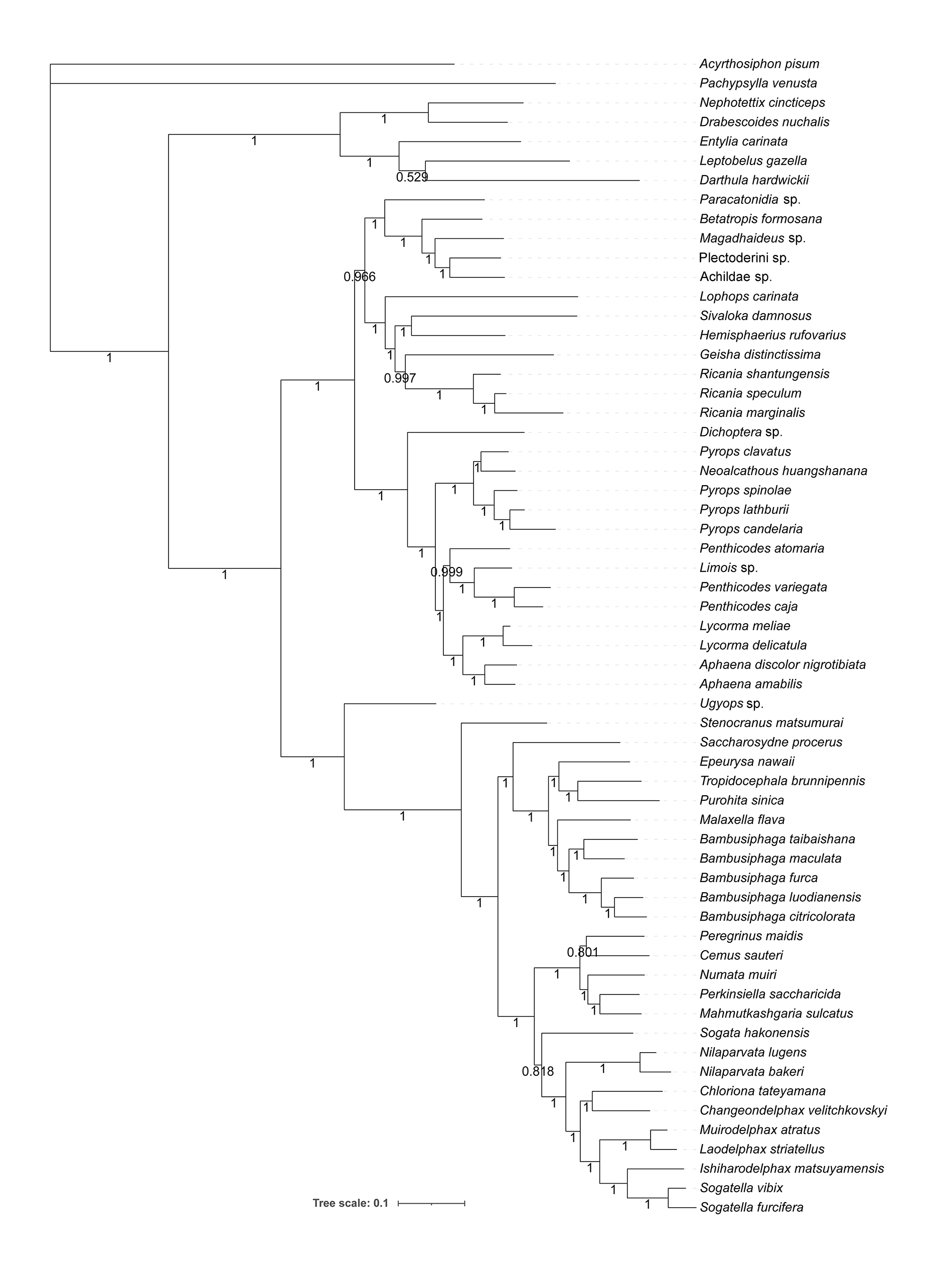

Supplement: Supplementary file 1 [file genes-12-01185-s001.zip › genes-1277800-supplementary/Supplementary Materials/Fig. S15 Phylogenetic tree BI_PCG12.jpg]

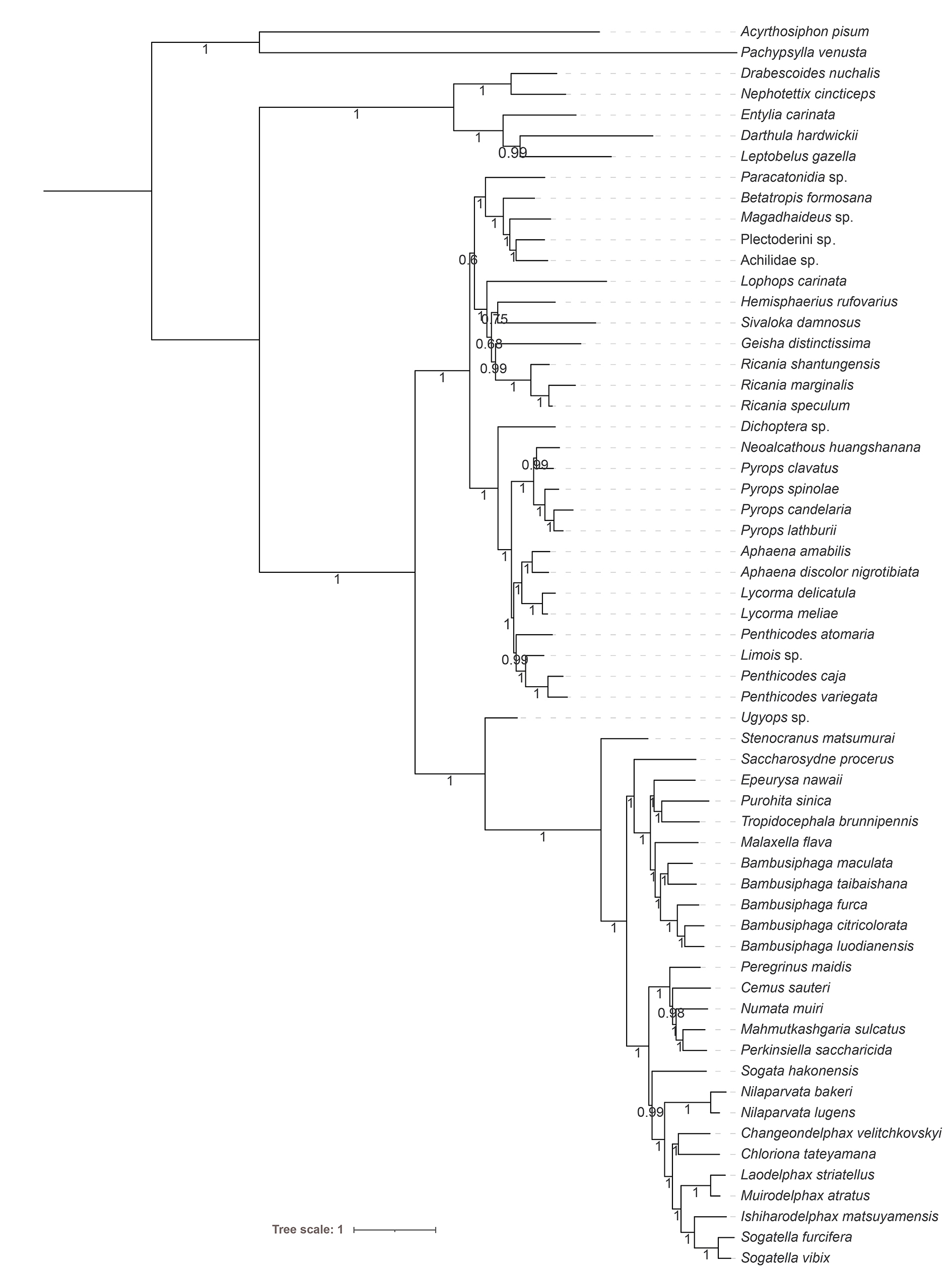

Supplement: Supplementary file 1 [file genes-12-01185-s001.zip › genes-1277800-supplementary/Supplementary Materials/Fig. S16-PCG123R.jpg]

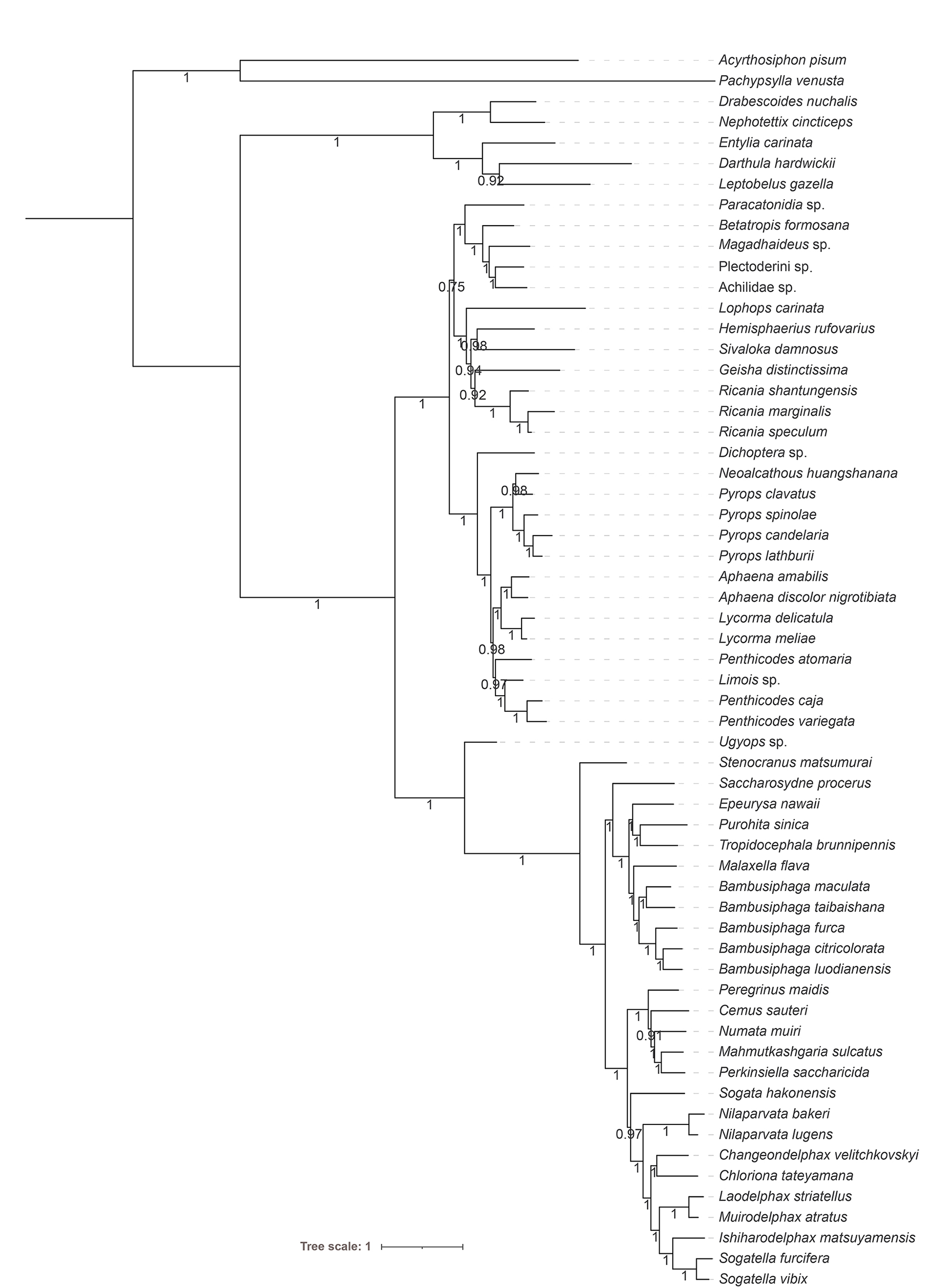

Supplement: Supplementary file 1 [file genes-12-01185-s001.zip › genes-1277800-supplementary/Supplementary Materials/Fig. S17-PCG123.jpg]

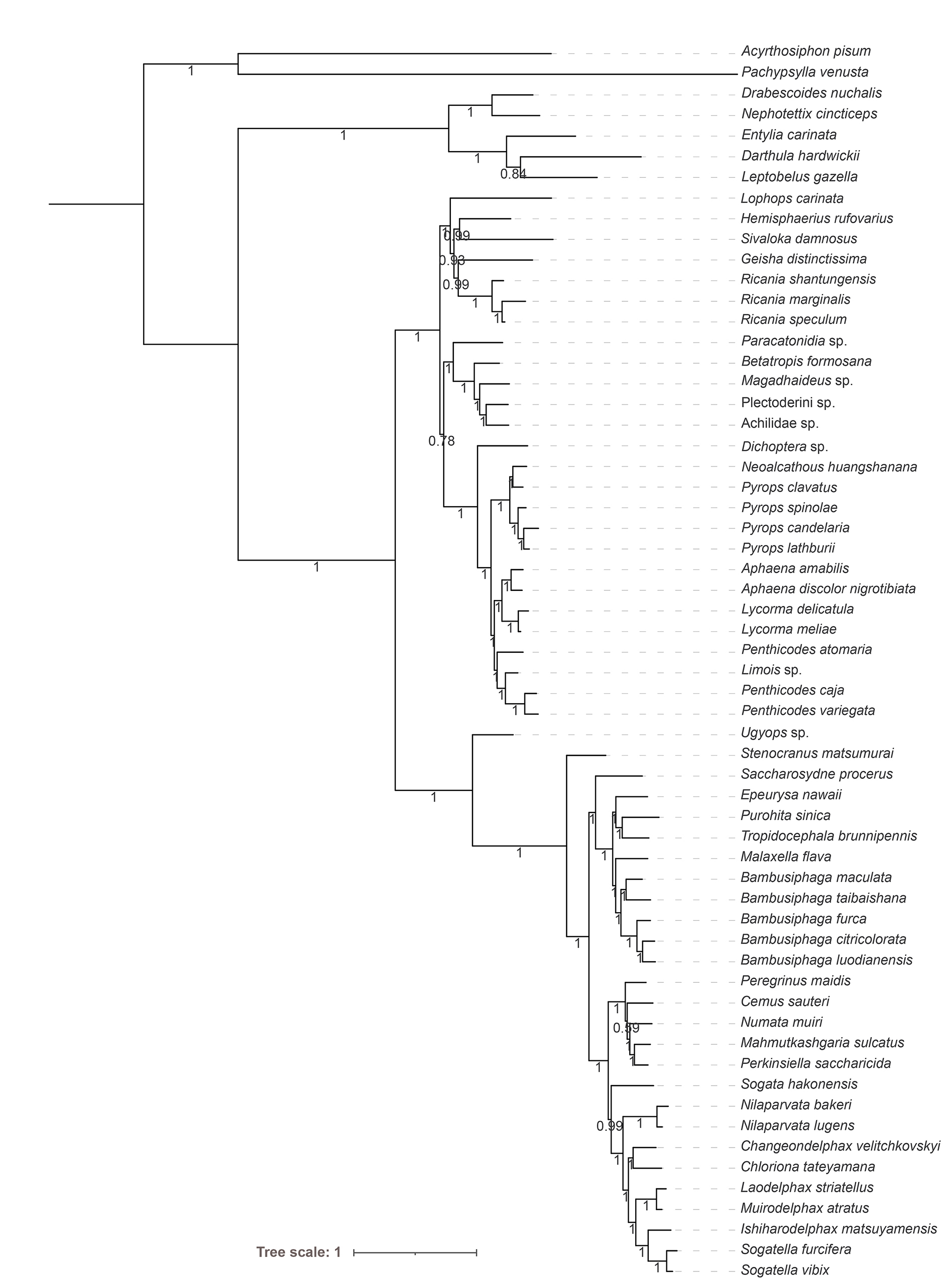

Supplement: Supplementary file 1 [file genes-12-01185-s001.zip › genes-1277800-supplementary/Supplementary Materials/Fig. S18-PCG12R.jpg]

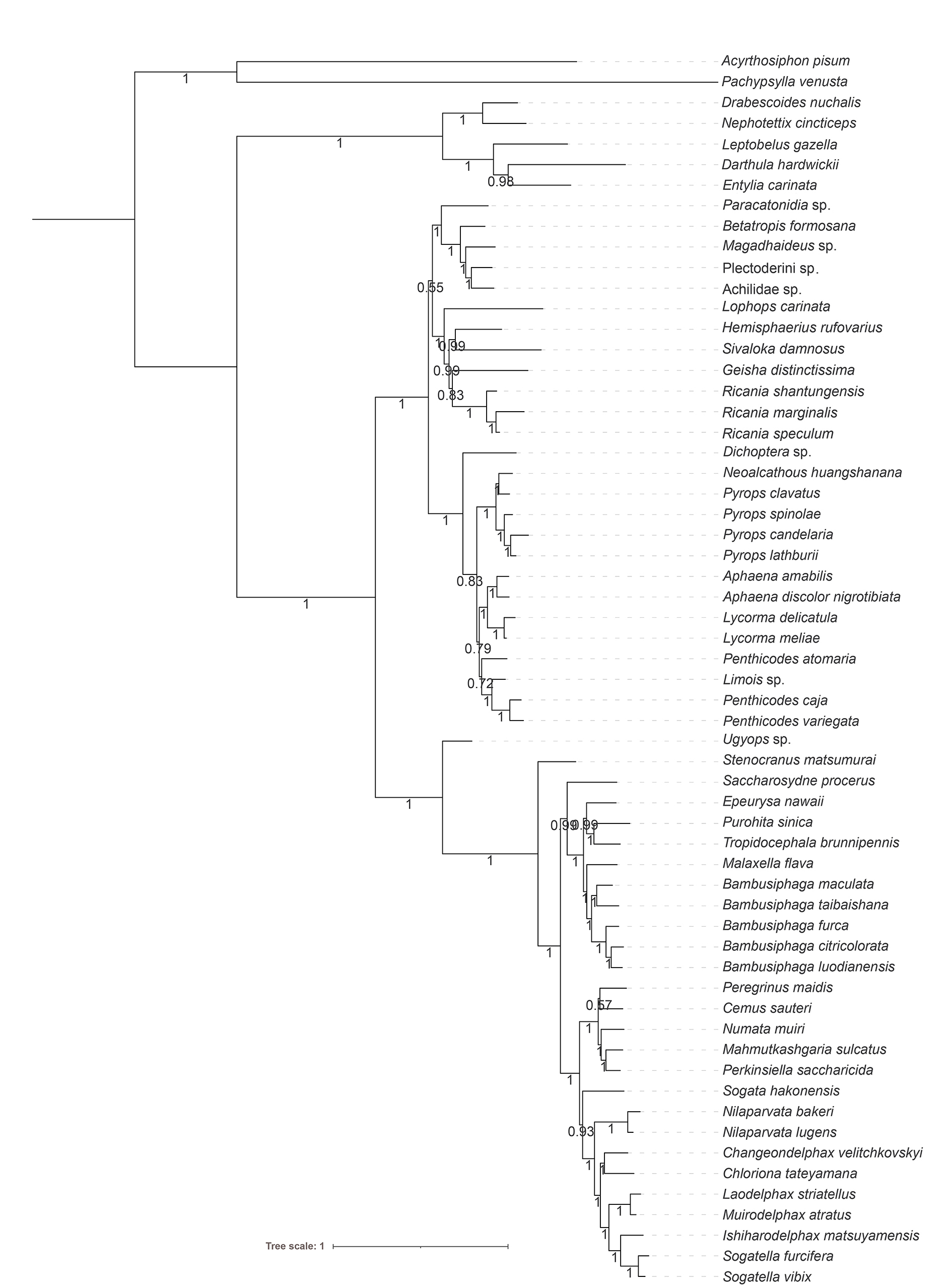

Supplement: Supplementary file 1 [file genes-12-01185-s001.zip › genes-1277800-supplementary/Supplementary Materials/Fig. S19-PCG12.jpg]

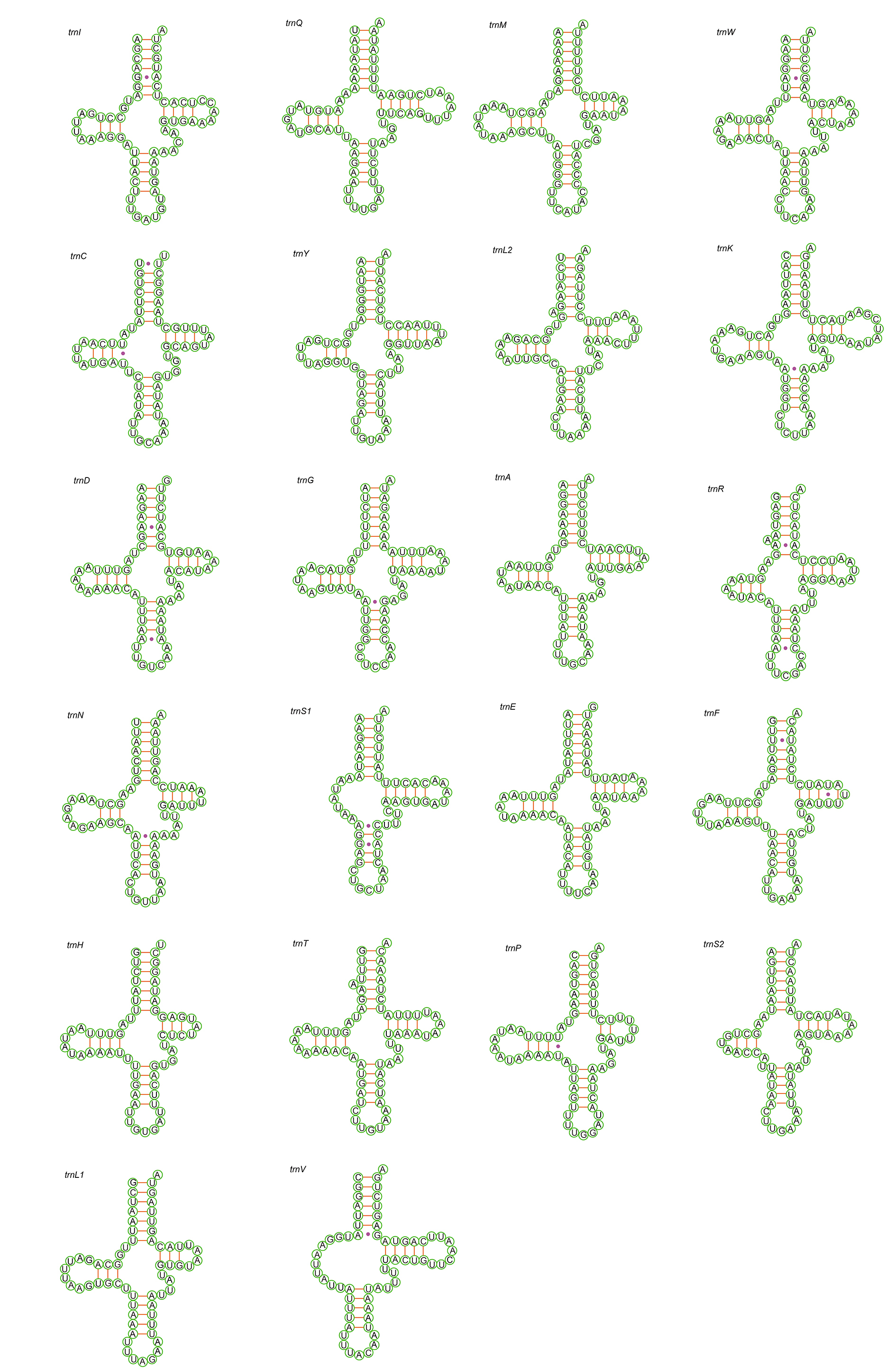

Supplement: Supplementary file 1 [file genes-12-01185-s001.zip › genes-1277800-supplementary/Supplementary Materials/Fig. S2 tRNA Limois sp.jpg]

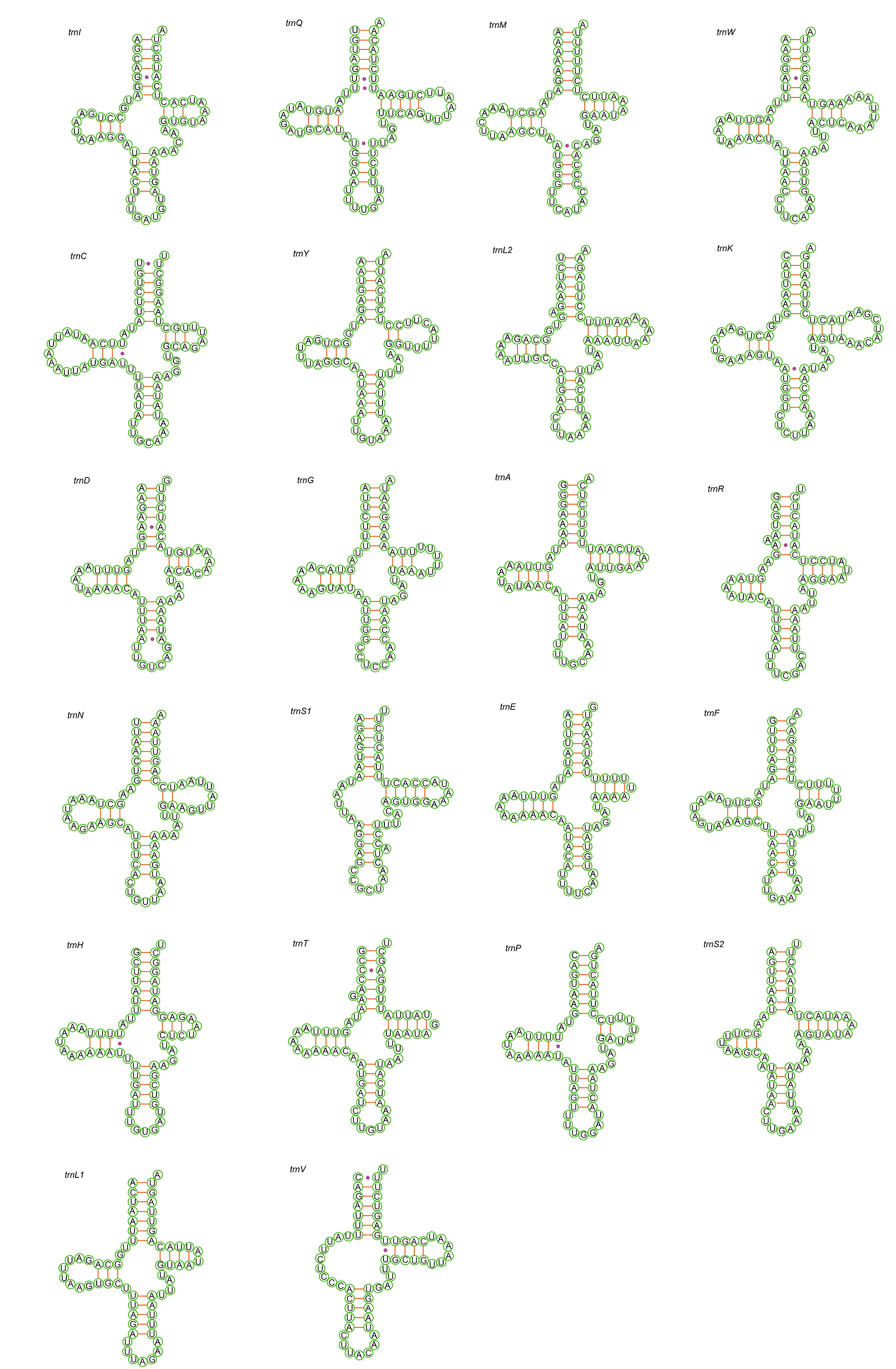

Supplement: Supplementary file 1 [file genes-12-01185-s001.zip › genes-1277800-supplementary/Supplementary Materials/Fig. S3 tRNA Neoalcathous huangshanana.jpg]

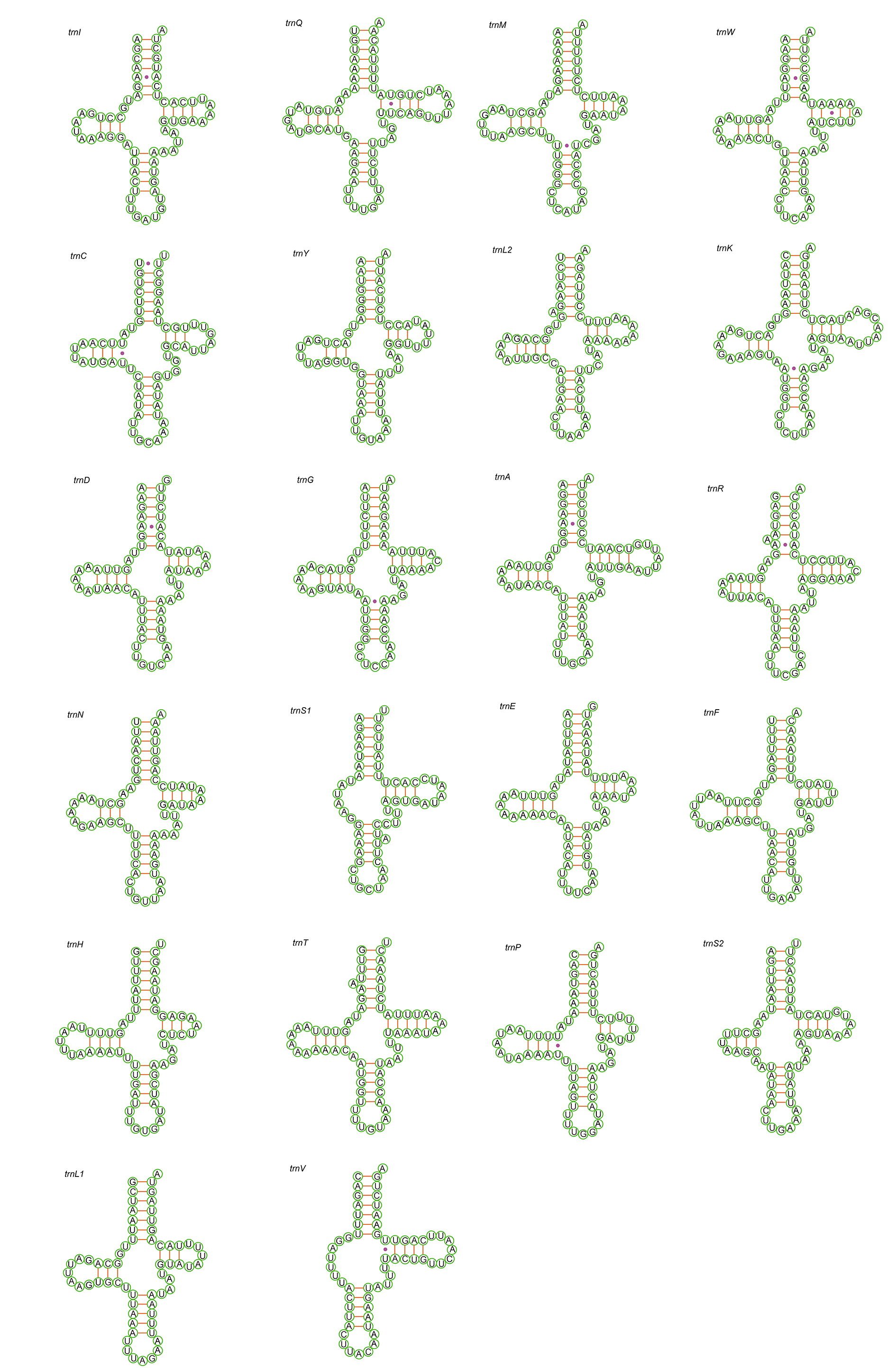

Supplement: Supplementary file 1 [file genes-12-01185-s001.zip › genes-1277800-supplementary/Supplementary Materials/Fig. S4 tRNA Penthicodes atomaria.jpg]

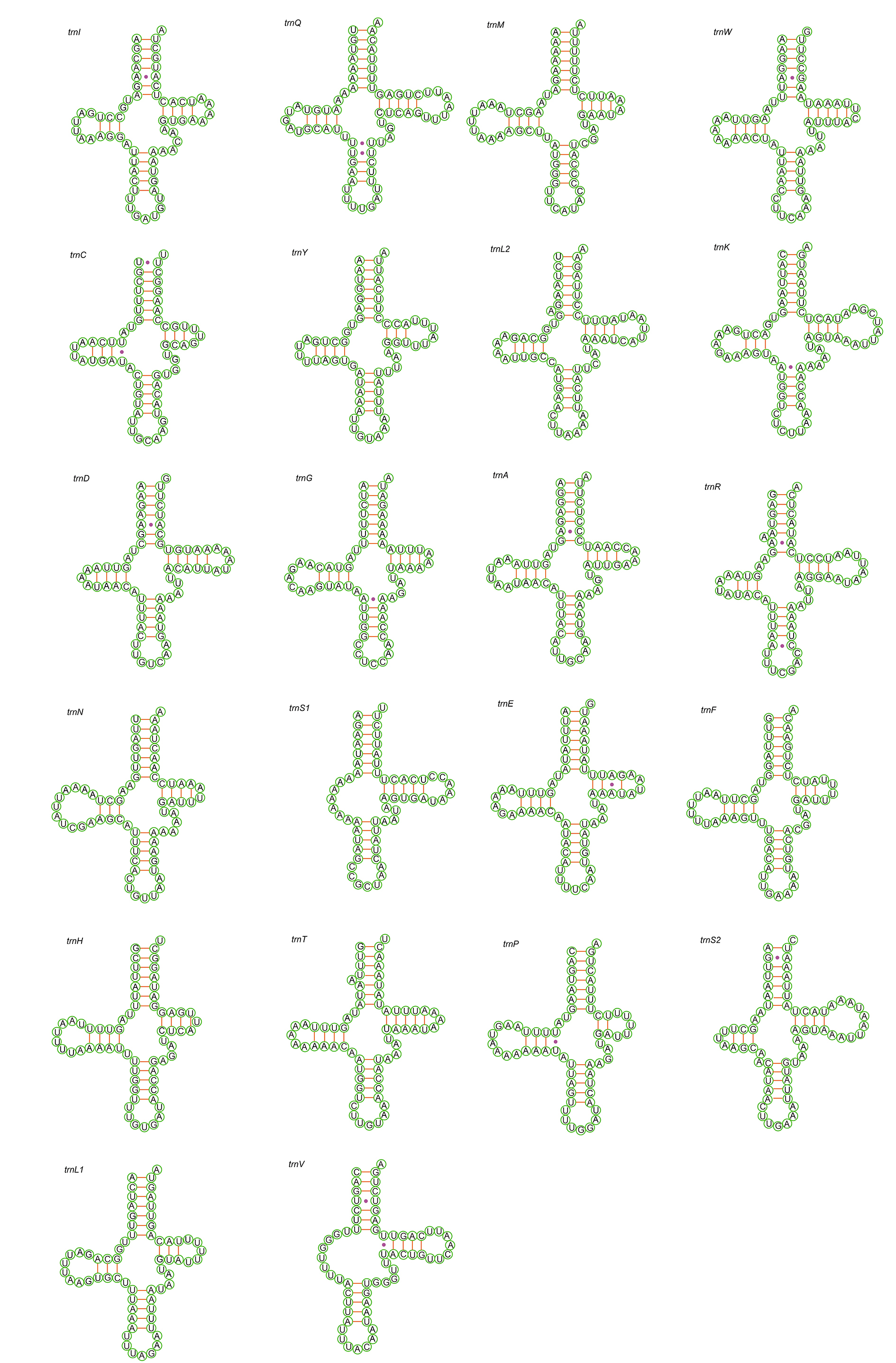

Supplement: Supplementary file 1 [file genes-12-01185-s001.zip › genes-1277800-supplementary/Supplementary Materials/Fig. S5 tRNA Penthicodes caja.jpg]

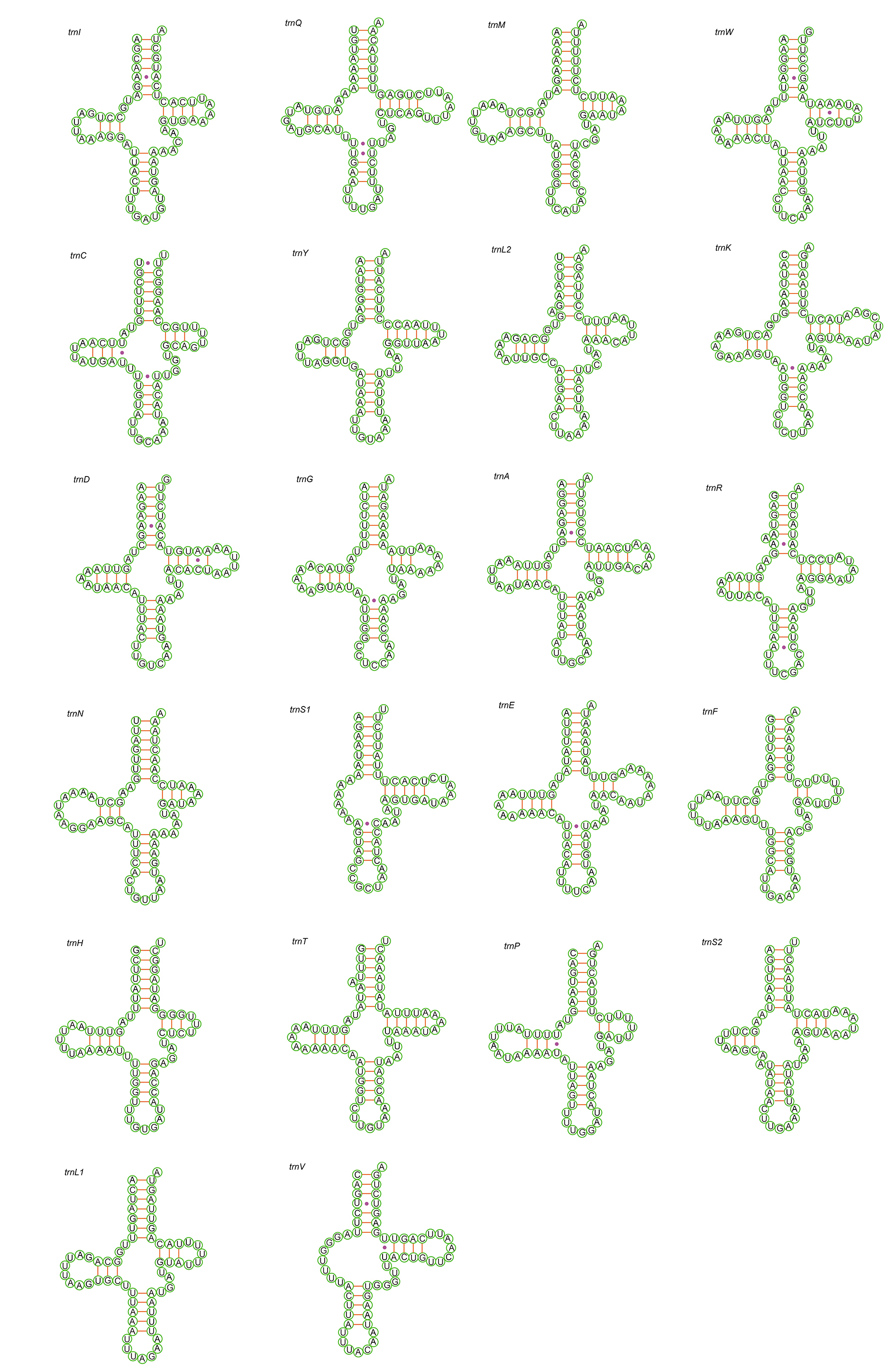

Supplement: Supplementary file 1 [file genes-12-01185-s001.zip › genes-1277800-supplementary/Supplementary Materials/Fig. S6 tRNA Penthicodes variegata.jpg]

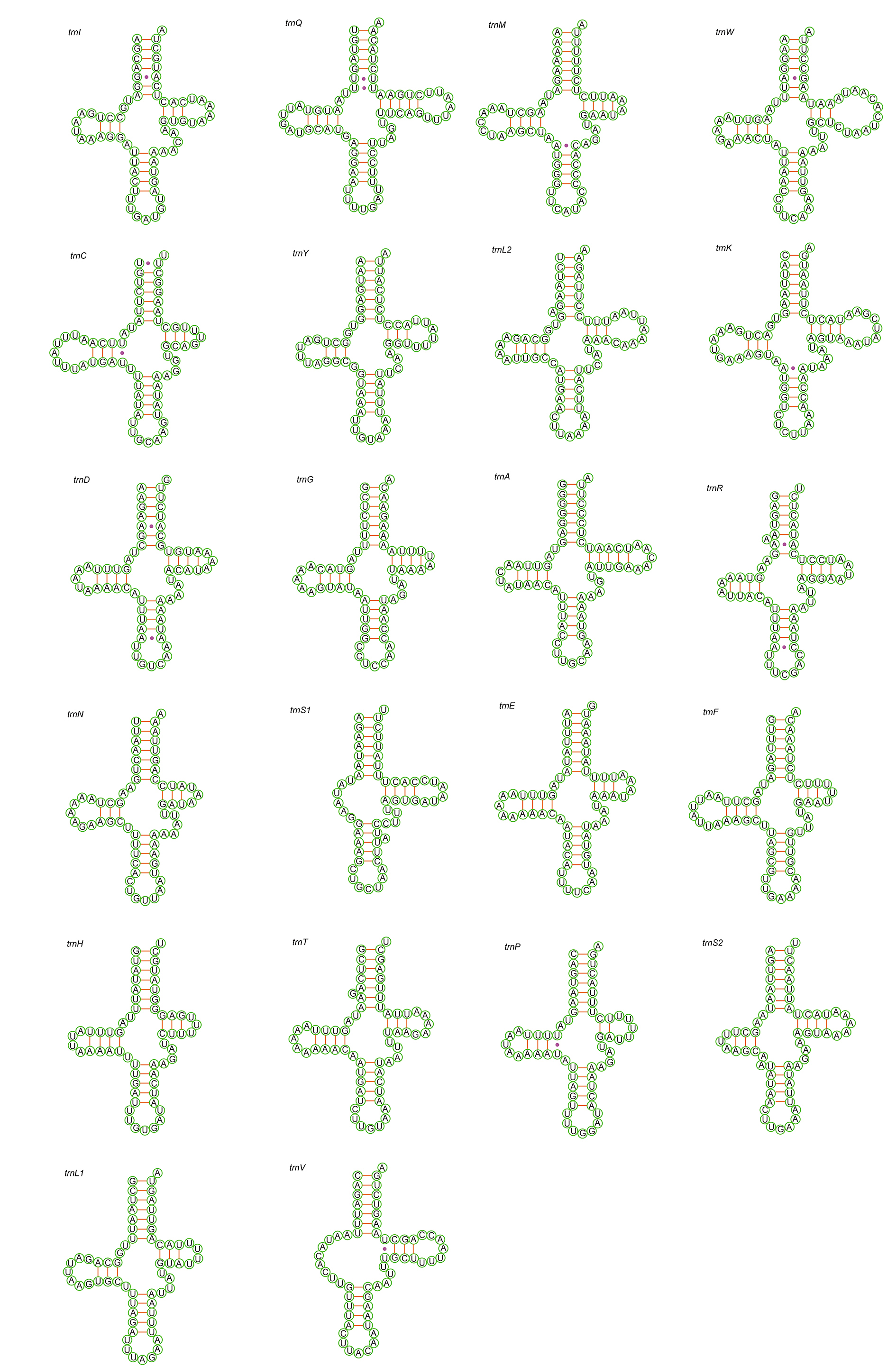

Supplement: Supplementary file 1 [file genes-12-01185-s001.zip › genes-1277800-supplementary/Supplementary Materials/Fig. S7 tRNA Pyrops clavatus.jpg]

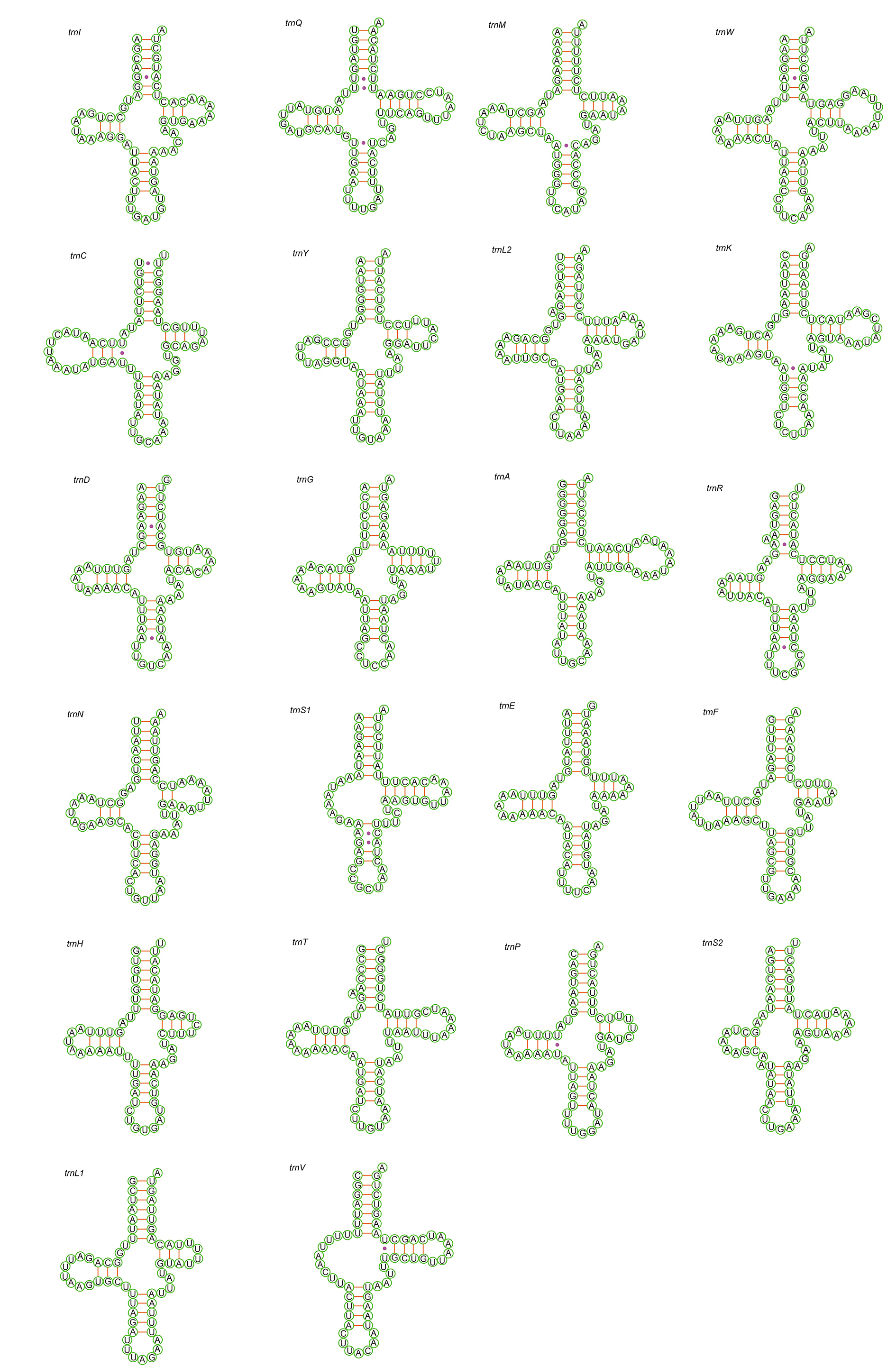

Supplement: Supplementary file 1 [file genes-12-01185-s001.zip › genes-1277800-supplementary/Supplementary Materials/Fig. S8 tRNA Pyrops lathburii.jpg]

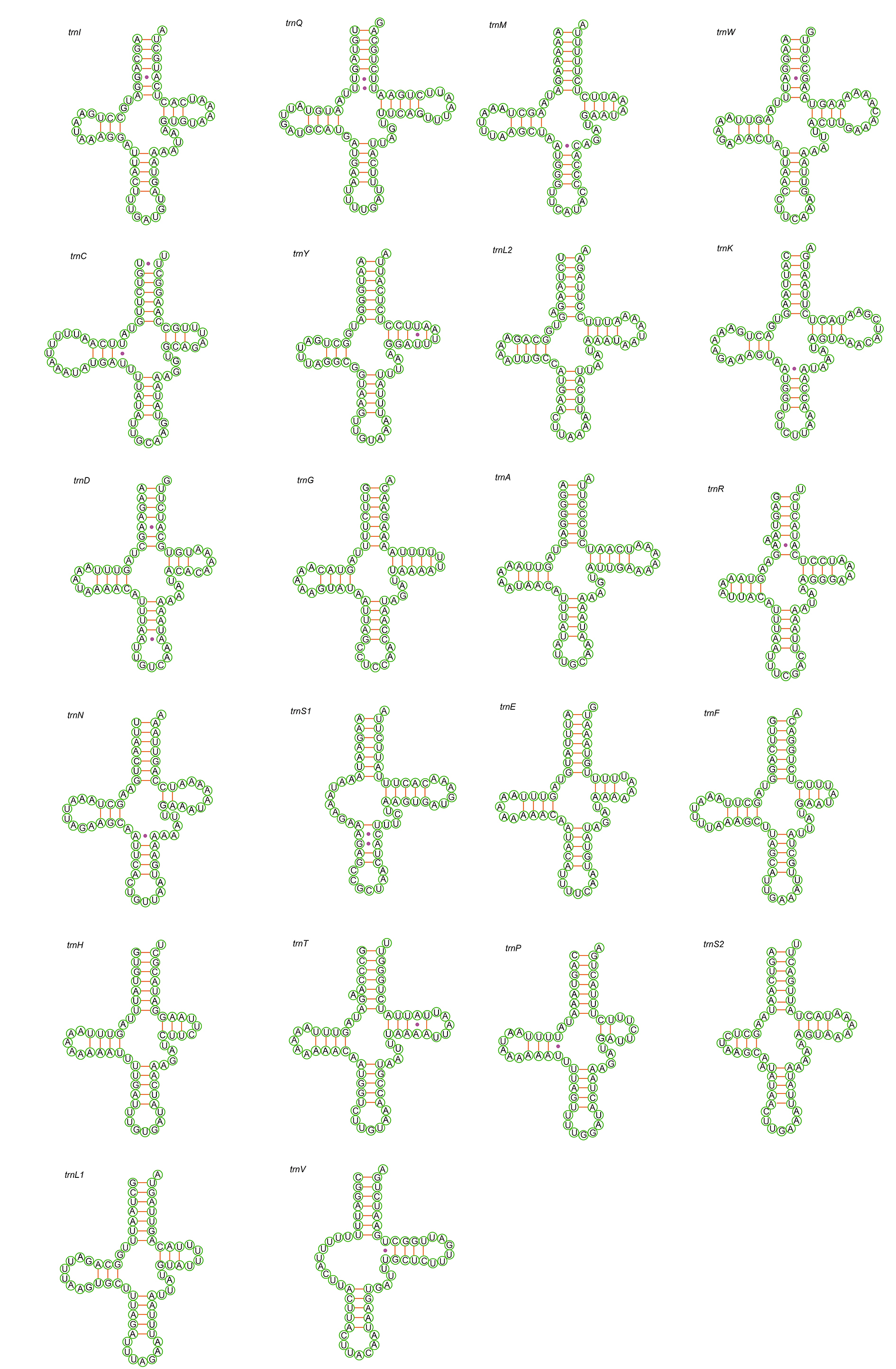

Supplement: Supplementary file 1 [file genes-12-01185-s001.zip › genes-1277800-supplementary/Supplementary Materials/Fig. S9 tRNA Pyrops spinolae.jpg]
